# Meeting Report: Structural Determination of Environmentally Responsive Proteins

**DOI:** 10.1289/ehp.8129

**Published:** 2005-07-13

**Authors:** Leslie Reinlib

**Affiliations:** Division of Extramural Research and Training, National Institute of Environmental Health Sciences, National Institutes of Health, U.S. Department of Health and Human Services, Research Triangle Park, North Carolina, USA

**Keywords:** Environmental Genome Project, gene–environment interactions, protein structure, structural biology

## Abstract

The three-dimensional structure of gene products continues to be a missing lynchpin between linear genome sequences and our understanding of the normal and abnormal function of proteins and pathways. Enhanced activity in this area is likely to lead to better understanding of how discrete changes in molecular patterns and conformation underlie functional changes in protein complexes and, with it, sensitivity of an individual to an exposure. The National Institute of Environmental Health Sciences convened a workshop of experts in structural determination and environmental health to solicit advice for future research in structural resolution relative to environmentally responsive proteins and pathways. The highest priorities recommended by the workshop were to support studies of structure, analysis, control, and design of conformational and functional states at molecular resolution for environmentally responsive molecules and complexes; promote understanding of dynamics, kinetics, and ligand responses; investigate the mechanisms and steps in posttranslational modifications, protein partnering, impact of genetic polymorphisms on structure/function, and ligand interactions; and encourage integrated experimental and computational approaches. The workshop participants also saw value in improving the throughput and purity of protein samples and macromolecular assemblies; developing optimal processes for design, production, and assembly of macromolecular complexes; encouraging studies on protein–protein and macromolecular interactions; and examining assemblies of individual proteins and their functions in pathways of interest for environmental health.

If the only tool you have is a hammer, you tend to see every problem as a nail.

—Abraham Maslow

Scientists are often no different from others in following the tendency of which Maslow spoke. Even in structural biology, a field that relies on complex mathematics, immense computing power, and cutting-edge technologies, investigations can tend to apply the specific skills at hand. However, with the emergence of genomic data and new technologies comes great challenges requiring multiple skills and scientific viewpoints. Participants at a recent workshop on structural determination of environmentally responsive proteins convened by the National Institute of Environmental Health Sciences highlighted some of these technologies but cautioned that the greatest strides will come only from seeing the problems as more than just nails.

Knowledge of the structures of individual proteins and how they fit together in macro-molecular complexes is critical to understanding function and to accelerating design of new molecular tools and more effective medicines. Although the Human Genome Project ignited an explosion of efforts to understand the blueprint of human health and disease, optimally applying the information flowing from linear gene sequences requires visualization of complete proteins in physiologically correct forms. Genomes have also been sequenced of varied pathogens and animals featured in laboratory experimentation, creating a high demand for the associated protein structures. To fulfill this demand, more advanced studies will be needed, taking advantage of the biophysics and understanding of posttranslational modifications that mandate protein folding and the ultimate form of the three-dimensional structures.

A long-term goal of these studies is to gain intimate insights into the function of protein complexes, enabling construction of ligand agonists and antagonists to assist efforts that will lead to clear mechanistic understanding and effective drugs. Among the recent successes in this arena are the structural resolutions of regions shared among the members of protein families involved in, for example, metabolism and detoxification. Key structural elements have been resolved of the components of cellular pathways—such as tyrosine kinases, G-protein subunits, and select apoptotic regulators—that mediate normal function and responses to environmental exposure and disease pathogens. Although many unique structures have been demonstrated in the population of 20,000–25,000 individual proteins that are predicted from the human genome, there is a widening gap between the expected numbers of genes and the consequent protein structures (International Human Genome Sequencing Consortium 2004). Including combinations and posttranslational modifications, about 100,000 gene products are predicted. In fact the number of sequences entered into public databases over the last several years is increasing at a much faster pace than the number of determined structures entered into the Protein Data Bank. Surprisingly, current estimates suggest that only 1,000–5,000 distinct, stable polypeptide chain folds exist in nature to accommodate the rich variety of domain structures. However, only about 700 of these distinct protein folds have been determined experimentally (Burley and Bonanno 2002). Further resolutions will be necessary to clarify the structural basis for function of the multiple components aligned in complexes and cell pathways of interest.

Accumulating evidence indicates that the structure of functional protein units is more complex than previously thought. It is becoming clear that the functions of many proteins occur as components of macromolecular complexes. Complexing may be required to fulfill a basic function (e.g., proper binding of tumor necrosis factor) or to synergize activity through, for example, altered binding affinities. BRCA-1, for example, interacts with a partner protein known as BARD1 (BRCA-1–associated ring domain). Although both BRCA-1 and BARD1 possess ubiquitin ligase activity, the combined complex is dramatically more active than either of the solitary proteins (Baer 2001). Synergies such as this may result from effects on binding affinities, efficiencies, and/or dynamics. Notably, these changes can occur in regions distal to protein active sites, suggesting that the impact of complexing may not be obvious from the examination of an individual side chain. To address the mechanisms of such effects, future studies will be needed on full-length proteins and macromolecular complexes, requiring an even greater set of skills and disciplines than in current practice.

Over the last decade, the National Institute of Environmental Health Sciences (NIEHS) has invested heavily in resequencing genes of interest in understanding the role of sequence variation in susceptibility to environmental perturbation. These environmentally responsive genes were chosen for their known or likely involvement in cellular pathways and diseases that involve environmental exposures, such as cancer, xeroderma pigmentosum, and Werner’s syndrome. As part of the NIEHS Environmental Genome Project (EGP), the resequencing and verification of about 550 genes was initiated on a set of 96 human samples obtained from the Coriell Resource Center (Coriell Institute for Medical Resources 2005; Wilson and Olden 2004). The EGP has recently decided to expand the data sets to an ethically defined panel and to explore more genes of interest to the research community. To date, approximately 280 genes have been completed, revealing more than 25,000 previously unknown polymorphisms (data available to scan and download at GeneSNPs http://www.genome.utah.edu/genesnps/). The data are useful for assessment of sensitivities based on single nucleotide polymorphisms (SNPs) in both population and basic science gene–environment projects. Individual SNPs may have significant effects on structure. Very elegant studies that indicate that intimate interactions of multiple regions within a protein contribute to overall efficiency have recently been reviewed (Tsigelny et al. 2004).

To better understand the relation of protein structural variation in environmentally responsive proteins to disease risk and resistance, NIEHS decided to appraise the state of the science and to explore optimal avenues for further research. Thus, on 26–27 April 2004, the Workshop on Structural Determination of Environmentally Responsive Proteins was convened at Snowbird Conference Center in Snowbird, Utah. The panel was composed of leading experts in the areas of crystallography, nuclear magnetic resonance, molecular biology, genomics, and environmental health sciences.

The workshop participants considered a variety of cutting-edge concepts and applications in structural biology. These included protein dynamics, protein–protein influences in macromolecular complexes, ligand responses, the impact of gene polymorphisms on predicted structures, and posttranslational modifications. Discussions also focused on the special requirements of studies of membrane proteins and the advantages of functionally based ligand design. A unique aspect of the workshop was the emphasis placed on environmentally responsive proteins and issues in environmental health sciences. For example, the structures discussed at the meeting included plasma membrane mercury transporters and P450 proteins. However, many of the topics and questions raised will no doubt be of general interest, and advances in these areas will likely be useful to a variety of research endeavors.

The workshop participants produced a set of ambitious, but practical, goals and prioritized recommendations that are discussed below.

## Recommendations

In considering how to optimize research resources in reaching specific goals in structural biology, the workshop participants strongly encouraged integrated, multidisciplinary programs that would maximally integrate basic science, computing, mathematics, and engineering. Although outstanding workers are found in all these fields, they approach their subject matter from disparate viewpoints and appear to speak different languages. It appears that communication and thus efficiency of operation are lacking among investigators in multiple, complementary areas. In accordance, the workshop participants recognized the need for cross-training among molecular biologists, geneticists, computer scientists, and mathematicians, especially among young investigators. Trainees with backgrounds in biochemistry, molecular biology, and physiology, as well as emerging areas, should be allowed to gain valuable skills in mathematics and computer science that could be applied to structural biology questions, particularly as they pertain to environmental health sciences.

The workshop participants recommended that interdisciplinary teams bring their talents to bear on gene products and pathways of interest to the environmental health research community. The workshop participants expressed the sentiment that such a focus was unlikely to come from the research community at large without leadership from the NIEHS and National Institutes of Health (NIH). Currently neglected areas highlighted for further investigation include—but are not limited to—complexes involving bioinorganic substances, for example, vanadate, aluminum fluoride, and borate; proteins in pathways influenced by environmental contaminants or dietary factors, such as endocrine disruptors; and DNA repair proteins.

Also, a variety of membrane proteins and membrane receptor complexes are considered to be understudied. Although proteins associated with biologic membranes comprise approximately 30% of the genome encoded peptides, only about 2% of the three-dimensional structures deposited in the Protein Data Bank—92 membrane proteins—are membrane proteins (White 2005; Zhou et al. 2004). The lower number of high-resolution three-dimensional structures makes homology modeling, in which existing structures are used as templates, difficult to apply to membrane proteins (Zhou et al. 2004). For environmental health studies, membrane proteins of interest include the components of stress signaling pathways and ion channels involved in the transport of xenobiotics. These include aryl hydrocarbon receptors, multidrug-resistance proteins, and transporters that facilitate uptake, metabolism, and clearance of environmental toxicants such as transporters of methylmercury and inorganic mercury. Membrane macromolecular complexes, in particular, present a number of laborious and complex tasks to resolve and are unlikely to be targets of interest for pharmaceutical companies attempting to bring drugs to market. These subjects could be timely for investigation by academic researchers.

After considering a variety of exciting new findings and technologies, the workshop participants recommended a set of specific scientific goals to enhance future research and contribute to the understanding of the structural and functional relationships of proteins and macromolecular complexes.

The workshop participants prioritized their suggestions into two groups. The recommendations with the highest priorities are discussed in further detail below. The secondary priorities were seen as later steps for investigation.

## Highest Priorities

- Support studies of structure, analysis, control, and design of conformational and functional states at molecular resolution for environmentally responsive molecules and complexes
- Promote understanding of dynamics, kinetics, and ligand responses
- Investigate the mechanisms and steps in posttranslational modifications, protein partnering, impact of genetic polymorphisms on structure/function, and ligand interactions
- Encourage integrated experimental and computational approaches.

## Secondary Priorities

- Improve the throughput and purity of protein samples and macromolecular assemblies (e.g., environmentally responsive membrane proteins)
- Develop optimal processes for design, production, and assembly of macromolecular complexes
- Encourage studies on protein–protein (macromolecule) interactions
- Examine assemblies of individual proteins and their functions in pathways of interest.

### Support studies of structure, analysis, control, and design leading to understanding of conformational and functional states at molecular resolution for environmentally responsive molecules and complexes.

There is a need to interface structural findings with biochemical outcomes, at both the *in vitro* and *in vivo* levels. The workshop participants pointed out that many talented investigators exploit a particular local skill, such as crystallography, but assistance from other scientific fields is needed to integrate these findings into a physiologic or even clinical model. Important questions permeate the field that require multiple viewpoints: How does structure lead to catalytic activity in protein kinases and other complexes of interest? How do protein dynamics play into the conformational changes that modulate function? How does misfolding lead to defective physiologic conditions?

Efforts to identify and determine the mechanisms and results of posttranslational modifications are inadequate. Gene sequencing provides a first step, but the ultimate amino acid chain can be modified radically in cellular processing. Efforts need to be redoubled to determine the final peptides. This point highlights the lack of studies performed under conditions that replicate the intracellular milieu. The final structure of a protein or complex of interest may differ significantly from that determined in ultrapure preparations. This is not a call to reintroduce impurities into samples, but rather to appreciate the influence of intracellular conditions on structure and function.

Continued focus is needed in determining molecular resolution that provides insights into interactions among proteins and in complexes. The complex need not be limited to proteins. The role of RNA, for example, appears to be underappreciated (Chien et al. 2004).

Strategies are needed to predict function from structure. Although investigators have learned much from biochemical considerations, structural resolution is frequently seen as an end point instead of a beginning for in-depth functional studies. To be most useful, structural determinations must be paired with models of how individual subunits interact with other molecules (Aloy et al. 2005). For example, DNA-associated proteins—DNA polymerases, glycosylases, and alkylases—are structurally diverse, but the relation of the known variations to function is not well understood. There are also a variety of proteins that fold into their “native state” on binding (De Lorenzi et al. 2004). Little is being done to discern how docking works in these cases. Docking methods attempt to maximally exploit all the available structural and chemical information possible from proteins, ligands, and protein–ligand complexes (van Dijk et al. 2005). “Guided docking’“ incorporates some degree of chemical information to actively guide the orientation of the ligand into the binding site (Fradera and Mestres 2004). Further work is needed to perfect such predictive models.

### Promote understanding of structural dynamics, kinetics, and ligand responses.

The workshop participants agreed that the fourth dimension must be considered to clarify the kinetics, specificities, affinities, and function of proteins. One limitation of current investigations is the fixed point in time in which structures are generally solved. The dynamics of a protein of interest or its interactions with neighboring proteins in its functional pathways are likely to be key to understanding the ultimate physiologic roles. Time- and temperature-dependent dynamics of domain fluctuations have been demonstrated in protein kinases and human estrogen receptors and are likely to be integral to the structure–function relationship of many other proteins. Thus, although “snapshots” of regional protein structures are accumulating, much less is known of their place in macromolecular complexes or with regard to time. Time dependence could be a critical factor influencing conformation and behavior of side chains and flexible regions. Importantly, an action in one domain could affect other, distally located sites, an event termed allostery (Kern and Zuiderweg 2004). Consideration of the time domain is often overlooked in structural studies but could be an essential part of the overall mechanism of biologic reactions. Quantitative time-dependent kinetic analysis would be expected to lead to new avenues that will produce working models of protein complexes and interactions in pathways. The results will elucidate the mechanisms of normal physiology, susceptibility, and disease.

The structures of crystallized proteins must be examined under varied conditions, not just in cell-free systems, to better understand the constraints, limits, and flexibilities of macromolecular complexes. For example, perturbing the system would likely reveal more information about binding specificity. This approach may not be applicable to SNP studies but would apply to design of ligands based on known DNA sequences.

Structures of membrane proteins pose unique problems, but the reward would seem to be worth the efforts. These proteins are often first responders to exogenous stimuli and mediate second messengers and other signaling processes, highlighting their importance in environmental health. Membrane proteins are often in relatively low abundance, and their study will require development of more robust expression systems to increase both yield and purity. Studies will also need region-specific labels that do not impede protein function of interaction with the membrane environment.

### Investigate the mechanisms and steps in posttranslational modifications, protein partnering, impact of genetic polymorphisms on structure/function, and ligand interactions.

The workshop participants indicated that three-dimensional structure is only a piece of the puzzle. Detailed atomic resolutions are also needed, as are insights into biochemical functions. To construct clear models relating structure with function, projects need to determine what the cellular function is for a protein complex and how that function relates to phenotype and susceptibility. Studies, then, may need to be performed on full-length proteins and under conditions replicating the natural milieu.

Proteins may need to be chosen that participate in complexes and interact with other proteins or nucleic acids. One example is the cold-shock proteins in bacteria. A major challenge is to make proteins amenable to study, especially for solving complex structures and assemblies. Better prediction is needed to determine the most promising protein fragments to study to optimize efficiency of time and cost. Much needed are new probes targeted to specific conformational states and individual steps of posttranslational modification. Binding agents are of particular interest for membrane proteins.

A compelling case was made for studies of the impact of SNPs on structure and function. Population studies are contributing a large amount of data linking SNPs with disease susceptibility. Combining these data with structural insights would increase the potential for improved mechanistic understanding and drug design. Tsigelny et al. (2004) provides a comprehensive overview of how multiple SNPs may affect P450 protein structures, such as aromatase. The authors suggest that visualizing the proteins allows focusing on likely sites controlling function, specificity for substrates, and the associated kinetics. Following this course, they suggest that the most significant impact would result if a particular SNP occurred in an area affecting “substrate recognition sites” or “substrate and product passage sites.” These types of value-added studies are encouraged because they indicate how structural insights, in providing new views of proteins, can lead to the design of novel ligands.

### Encourage integrated experimental and computational approaches.

Future investigations will require even more integration of information from diverse sources, especially in consideration of macromolecular assemblies. Fortunately, the technologies of crystallography and comparative modeling become very powerful when combined (for review, see Davis and Sali 2005). For example, it is impractical to measure the functional impact of every possible SNP at all positions in each protein of interest. Thus, prediction based on general principles of protein structure will save time and energy. There are, in fact, publicly accessible web servers to do just this, such asLarge Scale-SNP (Rachel and Sali 2005). The server accepts input specifications for a structure and a single amino acid mutation and outputs a prediction of whether or not the mutant protein is impaired, as well as associated justifications and altered features (Karchin et al. 2005; Pieper et al. 2004). The system has worked well for several known proteins and SNPs, such as human BRCA-1 domains. A key issue is to relate the results of the model with physiologic impact. It is not hard to imagine that physiologists and biochemists will be in greater demand to collaborate on structural biology projects, just as microarrays and genomics applications have become common in population studies.

Computational protein design also lends itself well to producing novel proteins and systems. Such designs can provide mechanistic insights into the workings of complexes. The vast number of possible protein structures based on the 20 common amino acids presents a dilemma for experimentalists. Function-based computer design allows for a multitude of parameters that can be tested *in silico*. A simple example is to examine open versus closed conformations in the absence or presence of ligands that are expected to bind based on conserved sequences. Proteins such as enzymes could also be designed that interact with environmental pollutants. For example, a theoretical protozyme that mediates ester hydrolysis by thioredoxin could be configured that would likely have measurable activity in reaction mechanisms (Bolon and Mayo 2001). The computer model would allow testing of limited mutagenesis that would indicate the degree of effectiveness of potential ligands.

## Summary

The last decade has seen an enormous expansion of insights into macromolecular structures indicating that proteins have a dynamic and complicated existence. Research in structural biology has exploded over the last decade, following a plethora of data on the genomes of humans and model organisms, and the advent of affordable computer power.

The NIEHS has an impressive history of supporting gene–environment studies. The NIEHS’s investments include extensive resequencing of > 250 human environmentally responsive genes, molecular epidemiology planning grants to form the basis for future projects, and a future program to resequence genes of interest in the laboratory rat. Building on these past initiatives, better understanding of the structure and, with it, molecular function of proteins of interest are of high priority. Along with improvements in the ability to predict and visualize protein structures come new challenges. One would like to understand not just how individual proteins operate, but also how they complex with other proteins, nucleic acids, and substrates. It follows that better ligands—and thus more promising drugs—could be constructed based on the three-dimensional images of the complex.

In a recent review of the complexities of DNA replication, Bruce Alberts (2003) worries that “a generation of biologists may have become lulled into believing that the essence of a biological mechanism has been captured, and the entire problem therefore solved” by construction and examination of two-dimensional cartoons of cell pathways. Alberts suggests that studies of biologic processes, such as DNA replication, will require collaborations of physicists, chemists, and structural and molecular biologists, the goal being to define the atomic structures of all the relevant proteins and the associated kinetics for the enzyme reactions. To this mix, we might add physiologists, biochemists, and epidemiologists to bring the scientific endeavor full circle to public health.

Ultimately, these steps, as fostered by NIH programs, such as the NIEHS EGP and the National Institute of General Medical Sciences Protein Structure Initiative, are expected to lead to improved research tools and more effective therapeutic drugs. It is important to look beyond “the hammer and the nail” for the best combination of techniques and strategies for the challenges ahead.

## Figures and Tables

**Table:** Participants in the NIEHS Workshop on Structural Determination of Environmentally Responsive Proteins

Patricia Jennings (chair)	Division of Physical Sciences, Chemistry, and Biochemistry University of California at San Diego La Jolla, California
Leslie Reinlib (co-organizer)	Division of Extramural Research and Training NIEHS Research Triangle Park, North Carolina
David Balshaw (co-organizer)	Division of Extramural Research and Training NIEHS Research Triangle Park, North Carolina
Thomas Ellenberger	Department of Biological Chemistry and Molecular Pharmacology Harvard Medical School Boston, Massachusetts
Traci Hall	Division of Intramural Research NIEHS Research Triangle Park, North Carolina
Dorothee Kern	Department of Biochemistry Brandeis University Boston, Massachusetts
Shohei Koide	Department of Biochemistry and Molecular Biology University of Chicago Chicago, Illinois
James Halpert	Departments of Pharmacology and Toxicology University of Texas Medical Branch Galveston, Texas
Stephen L. Mayo	Division of Biology California Institute of Technology Pasadena, California
Gaetano T. Montelione	Northeast Structural Genomics Research Consortium and Department of Molecular Biology and Biochemistry Rutgers, The State University of New Jersey Piscataway, New Jersey
John Norvell	National Institute for General Medical Sciences NIH Bethesda, Maryland
Stanley Opella	Center for Nuclear Magnetic Resonance Spectroscopy and Imaging of Proteins University of California at San Diego La Jolla, California
Robert London	Division of Intramural Research NIEHS Research Triangle Park, North Carolina
Andrej Sali	Departments of Biopharmaceutical Sciences and Pharmaceutical Chemistry California Institute for Quantitative Biomedical Research University of California San Francisco, California

## References

[b1-ehp0113-001622] Alberts B (2003). DNA Replication and recombination. Nature.

[b2-ehp0113-001622] Aloy P, Pichaud M, Russell RB (2005). Protein complexes: structure prediction challenges for the 21st century. Curr Opin Struct Biol.

[b3-ehp0113-001622] Baer R (2001). With the ends in sight: images from the BRCA1 tumor suppressor. Nature Struct Biol.

[b4-ehp0113-001622] Bolon DN, Mayo SL (2001). Enzyme-like proteins by computational design. Proc Natl Acad Sci USA.

[b5-ehp0113-001622] Burley SK, Bonanno JB (2002). Structuring the universe of proteins. Annu Rev Genomics Hum Genet.

[b6-ehp0113-001622] Chien CY, Xu Y, Xiao R, Aramini JM, Sahasrabudhe PV, Krug RM (2004). Biophysical characterization of the complex between double-stranded RNA and the N-terminal domain of the NS1 protein from influenza A virus: evidence for a novel RNA-binding mode. Biochemistry.

[b7-ehp0113-001622] Coriell Institute for Medical Resources 2005. Coriell Cell Repositories. Camden, NJ:Coriell Institute for Medical Resources. Available: http://locus.umdnj.edu/ [accessed 1 July 2005].

[b8-ehp0113-001622] Davis FP, Sali A (2005). PIBASE: a comprehensive database of structurally defined protein domain interfaces. Bioinformatics.

[b9-ehp0113-001622] De Lorenzi E, Giorgetti S, Grossi S, Merlini G, Caccialanza G, Bellotti V (2004). Pharmaceutical strategies against amyloidosis: old and new drugs in targeting a “protein misfolding disease”. Curr Med Chem.

[b10-ehp0113-001622] Fradera X, Mestres J (2004). Guiding docking approaches to structure-based designs and screening. Curr Top Med Chem.

[b11-ehp0113-001622] International Human Genome Sequencing Consortium (2004). Finishing the euchromatic sequence of the human genome. Nature.

[b12-ehp0113-001622] Karchin R, Diekhans M, Kelly L, Thomas DJ, Pieper U, Eswar U (2005). LS-SNP: large-scale annotation of coding non-synonymous SNPs based on multiple information sources. Bioinformatics.

[b13-ehp0113-001622] Kern D, Zuiderweg ERP (2004). The role of dynamics in allosteric regulation. Curr Opin Struct Biol.

[b14-ehp0113-001622] Rachel K Sali A 2004. LS-SNP: Large Scale Human SNP Annotation. San Francisco:University of California, San Francisco. Available: http://salilab.org/LS-SNP [accessed 27 June 2005].

[b15-ehp0113-001622] Pieper U, Eswar N, Braberg H, Madhusudhan MS, Davis FP, Stuart AC (2004). MODBASE, a database of annotated comparative protein structure models, and associated resources. Nucleic Acids Res.

[b16-ehp0113-001622] Tsigelny IF, Kotlovyji V, Wasserman L (2004). SNP analysis combined with protein structure prediction defines structure-functional relationships in cancer related cytochrome P450 estrogen metabolism. Curr Med Chem.

[b17-ehp0113-001622] Van Dijk AD, Boelens R, Bonvin AM (2005). Data-driven docking for the study of biomolecular complexes. FEBS J.

[b18-ehp0113-001622] White SH 2005. Membrane proteins of known structure. Irvine:University of California, Irvine. Available: http://blanco.biomol.uci.edu/Membrane_Proteins_xtal.html [accessed 20 June 2005].

[b19-ehp0113-001622] Wilson S, Olden K (2004). The environmental genome project: phase I and beyond. Mol Intervent.

[b20-ehp0113-001622] Zhou C, Zhang Y, Zhou Y (2004). Structure prediction of membrane proteins. Genomics Proteomics Bioinformatics.

